# Molecular profiling of single organelles for quantitative analysis of cellular heterogeneity

**DOI:** 10.1038/s41598-017-06936-z

**Published:** 2017-07-26

**Authors:** Andrey N. Kuzmin, Svitlana M. Levchenko, Artem Pliss, Junle Qu, Paras N. Prasad

**Affiliations:** 1 Advanced Cytometry Instrumentation Systems, LLC, 640 Ellicott Street – Suite 499, Buffalo, 14203 NY USA; 2 Institute for Lasers, Photonics and Biophotonics, University at Buffalo, State University of New York, Buffalo, NY 14260 USA; 3 College of Optoelectronic Engineering, Shenzhen University, Key Laboratory of Optoelectronic Devices and Systems of Ministry of Education and Guangdong Province, Shenzhen, Guangdong, 518060 China

## Abstract

Recent developments in Raman spectroscopy instrumentation and data processing algorithms have led to the emergence of Ramanomics - an independent discipline with unprecedented capabilities to map the distribution of distinct molecular groups in live cells. Here, we introduce a method for probing the absolute concentrations of proteins, RNA and lipids in single organelles of live cultured cells by biomolecular component analysis using microRaman data. We found significant cell-to-cell variations in the molecular profiles of organelles, thus providing a physiologically relevant set of markers of cellular heterogeneity. At the same cell the molecular profiles of different organelles can strongly correlate, reflecting tight coordination of their functions. This correlation was significant in WI-38 diploid fibroblasts and weak in HeLa cells, indicating profound differences in the regulation of biochemical processes in these cell lines.

## Introduction

Widespread heterogeneity between individual cells is a fundamental property of cellular populations, which arises from stochastic molecular interactions^1, 2^. Remarkably, the intercellular variations in gene activities, molecular structure and functions often play a decisive role in cellular fate by influencing proliferation, differentiation, and response to external stimuli^2–5^. Furthermore, cellular heterogeneity underlies the development of severe medical conditions including pathologic embryogenesis^6, 7^, neurodegenerative and psychiatric disorders^8^, skin diseases^9^, autoimmunity and immunodeficiency^10^, and cancer^11^.

There are a number of highly multiplex molecular recognition techniques currently available for the characterization of cell-to-cell variations. Most notably, these include single-cell transcriptomics, proteomics and metabolomics, depending on the analyte studied^12^. However, despite the heralded capability for high-throughput screening of biomolecules in the sample, “omic” techniques alone are not sufficient for comprehensive characterization of cellular heterogeneity. One major shortcoming of current techniques is that they involve cell destruction, meaning that analysis can be performed only once and at discrete time point. In addition, whole cell molecular datasets are inherently misleading as the properties and functions of biomolecules often depend on their intracellular location; therefore molecular profiling of organelles would be more meaningful. However, the sensitivity of relevant organelle-centered techniques is not at a single-cell level yet, although this direction has been actively pursued in recent years^13–15^. To date there are no established methods for non-invasive molecular analysis of different organelles that can be used to investigate the dynamic nature of cell-to-cell variations, their emergence, propagation in time, and the resulting impact on cellular fate.

Remarkably, the capabilities of conventional molecular recognition tools can be significantly expanded with biophotonic non-invasive approaches, which provide high 3D resolution and label-free sensing of subcellular molecular environment. Among the most elegant tools, Raman spectroscopy combined with our recently developed algorithms for biomolecular component analysis (BCA)^16^ has led to the emergence of a radically different branch of omics disciplines. Raman spectra are produced by the inelastic scattering of light and serve as chemical fingerprints of the samples. One distinct strength of the microRaman-BCA approach is the capability to selectively recognize and measure absolute concentrations of basic classes of biomolecules such as proteins, lipids, RNA, DNA and saccharides in the studied samples. Although the molecular selectivity of Raman spectroscopy is not nearly as high as in conventional molecular recognition techniques, it does not involve cell destruction, thus uniquely enabling the monitoring of the local molecular environment in live cells and their subcellular structures. MicroRaman-BCA is the only tool, which enables non-invasive concentration profiling of diverse cellular compartments such as nucleoli^16^, lysosomes^17^, mitochondria^18, 19^ and also capable of identifying the characteristic changes in the physico-chemical properties of organelles during various cellular processes^18, 20^. The sensitivity and molecular selectivity of microRaman-BCA is sufficiently high to investigate spontaneous fluctuations of ribosomal RNA synthesis in the nucleoli of live cultured cells^21^ and to identify changes in proteins conformation in the process of cell fixation^22^. Moreover, data obtained by microRaman-BCA are similar to those estimated by an alternative indirect technique that is based on the correlation between the analyte concentration and the local refractive index^23^. Considering the distinct capabilities and growing popularity of Raman spectroscopic approaches, we project the emergence of a new discipline investigating the biochemical composition of cells and tissues - Ramanomics.

In this study, we applied the non-invasive capabilities of microRaman technique for molecular profiling of major cellular organelles in live WI-38 diploid fibroblasts and HeLa cancer cells. Specifically, we measured the concentrations of protein, RNA and lipid molecular groups in nucleoli, endoplasmic reticulum (ER) and mitochondria at a single organelle level. Surprisingly, we observed significant (up to 10 times) variations in the organelle-specific molecular concentrations in both cell lines. These variations were far from random; for each organelle, we found a correlation between the concentrations of RNA and proteins. Furthermore, in WI-38 cells our study unravels a remarkably strong connection between the molecular profiles in ER and nucleoli from the same cell, which suggests a high coordination between these organelles functions.

However, HeLa cells failed to produce a similar correlation. We suggest that this lack of correlation is due to oncogenic deregulation of ribosome synthesis and translation control, leading to uncoordinated changes in molecular profiles of nucleoli and ER in HeLa cells^24, 25^.

Finally, our study indicates that the molecular content of cellular organelles can rapidly change during cell growth. By monitoring the molecular composition of nucleoli in the same cell, we report that concentrations of RNA and proteins continuously change as a function of time. These changes apparently represent fluctuations in the nucleolar synthesis of ribosomes. At the same time, nucleoli of HeLa and WI-38 cells demonstrated different dynamics of fluctuations, with WI-38 cells producing less frequent, but more significant changes as compared to HeLa cells.

Thus, our findings unravel a complex correlation that exists between the molecular composition of different organelles of the same cell. Moreover, this study proposes macromolecular profiles of cellular organelles as novel set of quantitative markers for assessment of cellular physiologic state and identification and characterization of cellular heterogeneity.

## Results

### *In situ* molecular profiling of single cellular organelles in live cultured cells

These studies were performed in HeLa and Wi-38 cell lines. Our initial objective was to measure the concentrations of proteins, RNA and lipids, the most abundant types of cellular macromolecules and investigate potential variations between the different cells growing in the same cell culture. For molecular profiling, we selected three prominent organelles i.e. nucleoli, ER and mitochondria, for their fundamental roles in gene expression and metabolism.

**Figure 1:**
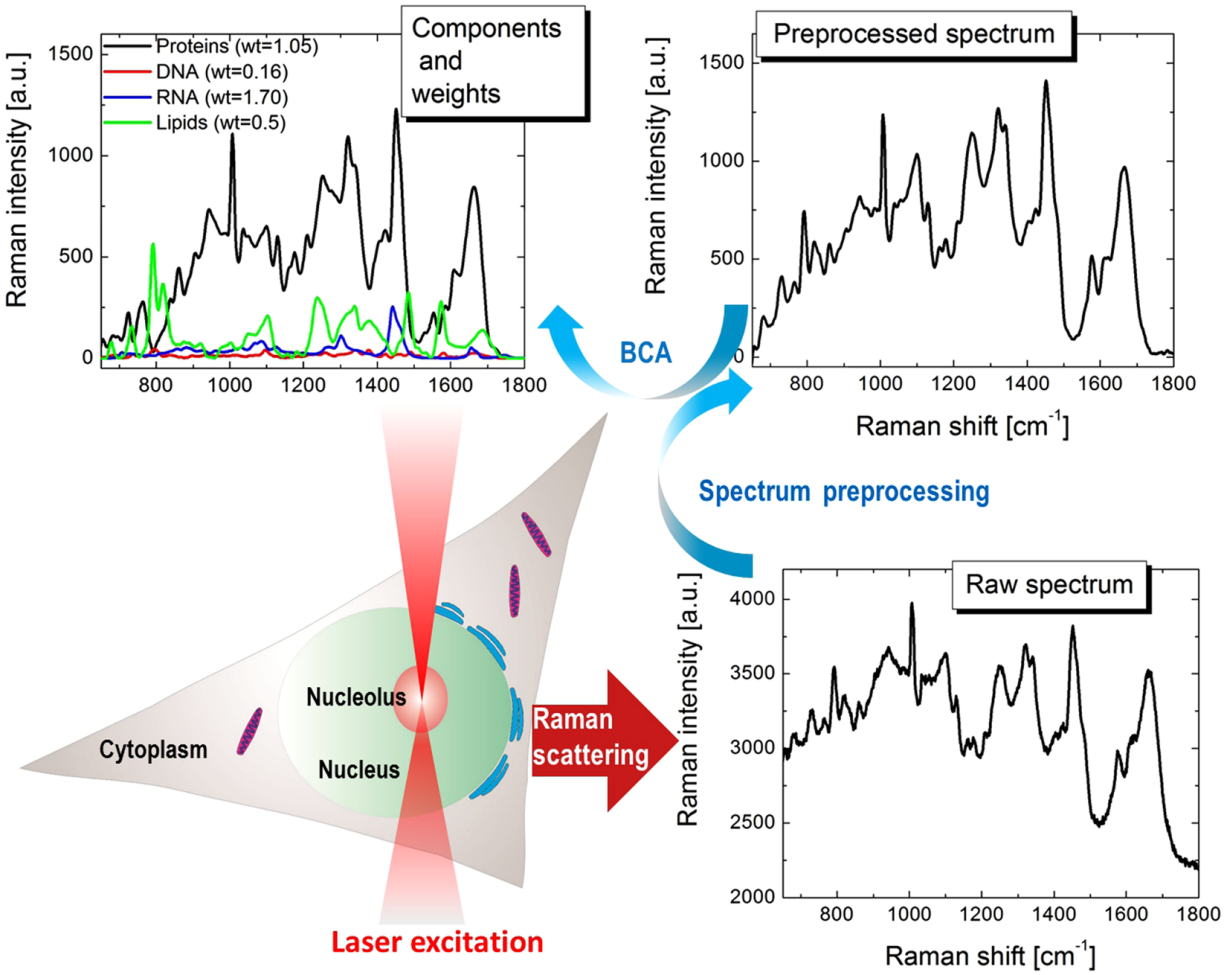
Schematics of microRaman-BCA approach for molecular profiling of cellular organelles: measurement of organelle’s spectrum, preprocessing and decomposition of spectrum to biomolecular components with concentration weights (proteins, DNA,RNA, lipids).

To measure concentrations of macromolecules *in situ* we employed the microRaman-BCA technique previously developed by our group^21, 22, 26^. In live cells the ER and mitochondria were visualized by organelle specific fluorescent probes, and nucleoli were identified by transmitted light imaging. The excitation laser was overlapped with the labeled organelles, and the Raman spectra were measured (Fig. 1). Only one spectrum per cell was acquired to avoid any potential phototoxicity. Using this approach, we acquired organelle-specific spectra for each cell line. The spectra were subsequently processed by a BCA algorithm, which includes background subtraction, baseline correction, and nonlinear least squares curve fitting to identify and quantify the contributions made by RNA, lipids and proteins. The processed spectra were used to determine the macromolecular concentrations (Fig. 1). In all the studied organelles, the contributions of proteins, RNA and lipids could easily be identified. The spectral weights associated with these compounds were well above the detection threshold (≥0.1), which enabled confident measurements of concentrations of these macromolecules in the studied organelles. In addition, spectra periodically contained trace contributions of other types of biomolecules such as DNA in nucleoli and mitochondria, and saccharides; however, these Raman signals were too weak for any reliable analysis.

**Figure 2:**
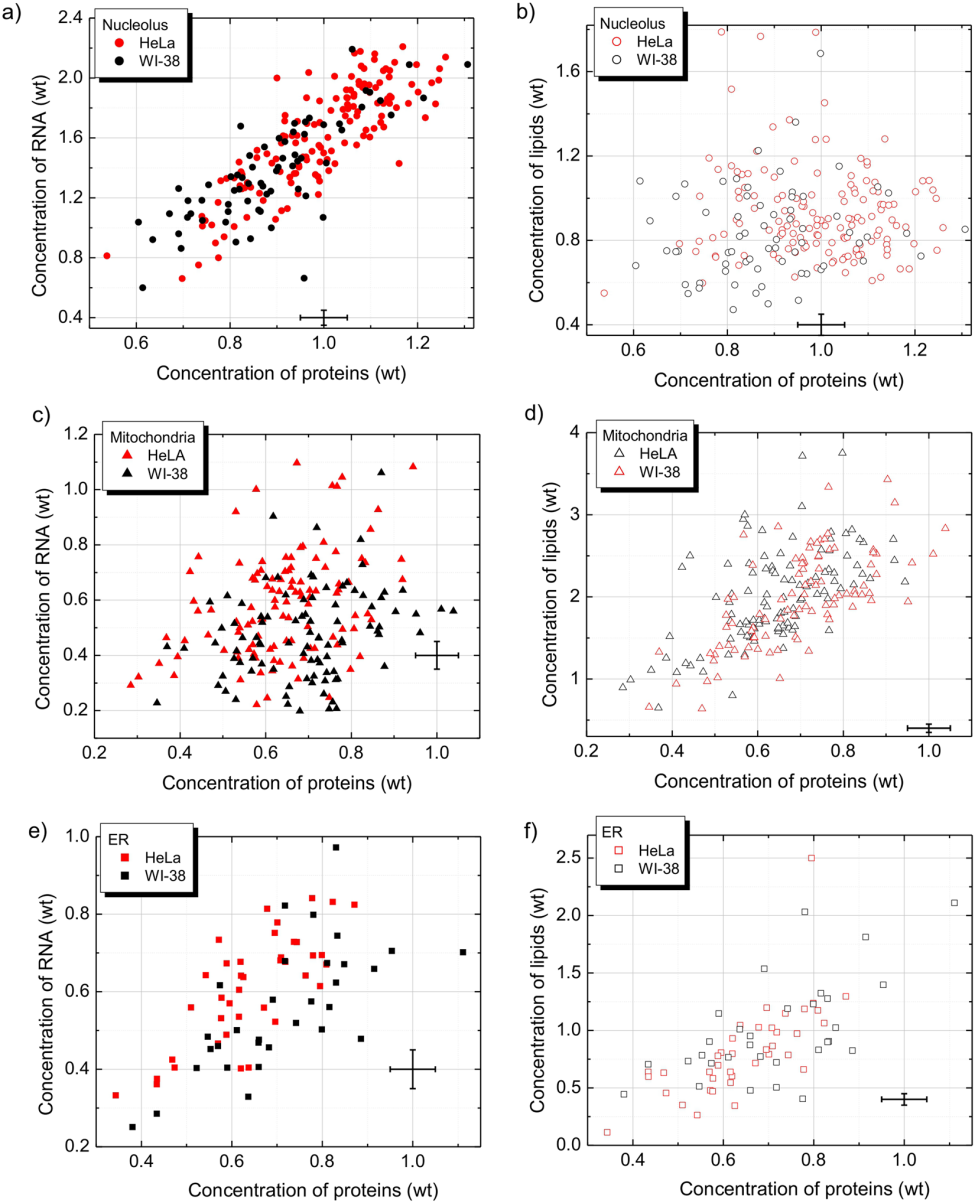
Concentrations of lipids and RNA versus concentrations of proteins in the nucleoli (**a**,**b**), mitochondria (**c**,**d**) and ER (**e**,**f**). Plots represent a distribution of molecular profiles in single cells. Bold symbols – RNA vs proteins, open symbols – lipids vs proteins, red symbols – WI-38, black symbols – HeLa. Standard deviations are shown as error bars. Concentration weight unit corresponds to 100 mg/ml for proteins, 20 mg/ml for RNA and lipids.

Obtained organelle-specific molecular concentration profiles indicate remarkably broad cell-to-cell variations for both WI-38 and HeLa cell lines (Fig. 2). In the nucleoli, the concentration of proteins varied ~50 mg/ml to ~120 mg/ml, concentration of RNA from ~10 mg/ml to ~45 mg/ml, and concentration of lipids from ~10 mg/ml to ~35 mg/ml (Fig. 2a). ER contained from ~20 to 120 mg/ml of proteins, from ~5 mg/ml to 20 mg/ml of RNA and 5 to 60 mg/ml of lipids (Fig. 2b). Finally, the molecular makeup of mitochondria includes ~35 mg/ml to ~110 mg/ml of proteins, from ~5 mg/ml to 20 mg/ml of RNA and from ~3 to 40 mg/ml of lipids in different cells (Fig. 2c).

To investigate whether the molecular makeup of organelles significantly differs in primary diploid WI-38 cells and Hela cells, we performed a statistical analysis using one way ANOVA with standard significance level 0.05. Details of this analysis are provided in Table S1 (Supplementary Materials).

Interestingly enough, despite a significant overlapping between the concentrations in WI-38 cells (black symbols) and HeLa cells (red symbols), there is a significant difference between the concentration profiles in these two cell lines. In the nucleoli of HeLa cells mean concentrations of proteins, RNA and lipids were 10–15% higher than the corresponding concentrations in the nucleoli of WI-38 cells (Table S1). In the ER of HeLa cells, there was ~10% higher concentration of proteins, while the mean concentrations of RNA and lipids were similar. Finally, in the mitochondria of HeLa cells the concentrations of proteins and RNA were ~10% and ~20% higher than in mitochondria of WI38 cells, respectively. No significant differences were found in the mitochondrial lipid signal between both cell lines.

**Figure 3:**
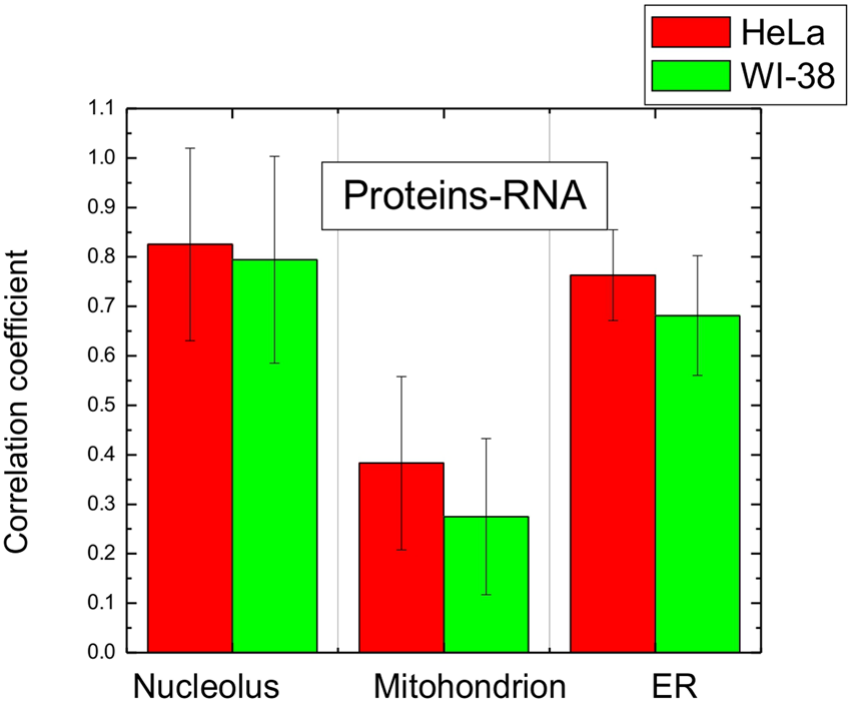
Pearson correlation coefficients between the concentrations of proteins and RNA of the same organelle - nucleoli, mitochondria and ER.

Next, we examined whether the concentration of RNA and proteins in the same organelle mutually correlate or independently vary. To examine this we processed the data sets for each studied organelle using a pairwise correlation analysis. The correlation levels were quantitatively estimated by the Pearson linear correlation coefficients, ranging from −1 to 1, wherein −1 is the maximum negative correlation, 0 is the absence of correlation, and 1 is the maximum positive correlation. This analysis revealed a strong correlation between RNA and proteins concentrations in the nucleoli and ER. The Pearson correlation coefficients were approximately at the same level (~0.80) in these organelles for both HeLa and WI-38 cell lines (Fig. 3). In mitochondria, the Pearson coefficients between RNA and proteins concentrations were at ~0.25 and ~0.35, indicating weak to moderate levels of correlation for WI-38 and HeLa cells correspondingly.

These findings suggest that despite very significant variations in the absolute concentrations of biomolecules found in organelles of different cells (Fig. 2a–c), the ratios between organelle macromolecules remains relatively constant in different cells growing in the same culture.

### Identifying the correlation between the molecular profiles in different organelles of the same cell

**Figure 4:**
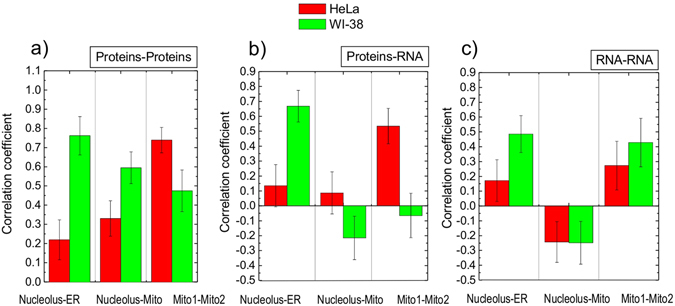
Pearson correlation coefficients between the concentrations of proteins and RNA in two organelles of the same cell, as indicated.

The synthesis of ribosomes in nucleoli and synthesis of proteins in ER are two intense and functionally interconnected processes of gene expression. Strikingly, little is known about the temporal coordination of these activities, despite the fundamental significance of such coordination for cellular regulation. To address this problem we compared the macromolecular profiles between nucleoli and ER. In these experiments, Raman spectra were sequentially acquired in nucleoli and ER in the same cell. Next, the Pearson coefficients were calculated to determine pairwise correlations for protein-protein, RNA-RNA and protein-RNA concentrations between each organelle. This simple approach unraveled very significant differences between the molecular organization of WI-38 and HeLa cells. In WI-38 cells we found a strong correlation between the concentrations of proteins and RNA in nucleoli and the corresponding concentrations in the ER of the same cell. The Pearson coeficients for protein-protein and protein-RNA concentrations in the ER and the nucleoli were at ~0.75 and ~0.70, respectively (Fig. 4a,b). For RNA-RNA concentrations the Pearson coeficient was at ~0.50 indicating a moderate to high correlation (Fig. 4c). In contrast, we found no significant correlation between the molecular content of the ER and the nucleoli in HeLa cells. None of the Pearson coeficient for protein-protein RNA-RNA and protein-RNA concentrations exceeded ~0.20. Similary, the Pearson coeficient for protein-protein concentrations in the nucleoli and mitochondria were ~0.60 and ~0.30 in WI-38 and HeLa cells, respectively (Fig. 4a). WI-38 cells also had higher correlation between the RNA concentrations in these two organelles.

In addition, HeLa cells demonstrated a higher protein-protein (0.74 versus 0.45) and protein-RNA (0.54 versus ~−0.10) correlation between two different mitochondria of the same cell (Fig. 4a,b). Both cell lines showed absence or negative correlation between the and protein-RNA concentrations in mitochondria and nucleoli (~0.10 in HeLa and ~−0.20 in WI-38).

### Dynamic changes in the molecular makeup of nucleoli in WI-38 and HeLa cells

**Figure 5:**
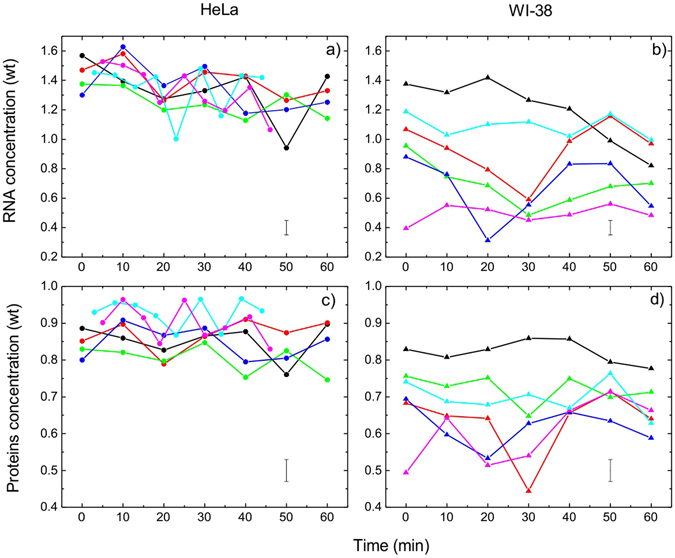
Dynamics behavior of RNA (**a**,**b**) and proteins (**c**,**d**) concentrations in 6 single nucleolus of HeLa (**a**,**c**) and (WI-38 (**b**,**d**) cells. Standard deviatios are shown as error bars in lower sector of each graph. Concentration weight unit corresponds to 100 mg/ml for proteins and 20 mg/ml for RNA.

To expand our characterization of macromolecular cell heterogeneity, we studied the dynamic behavior of organelle profiles, i.e. how the molecular profiles in the same organelle may change over time. In these experiments, time-sequenced Raman spectra were acquired in the same nucleoli over a ~60 minute period with ~10 minute intervals using the protocol from our previous study^21^. We found that nucleolar concentrations of proteins and RNA noticeably change between the subsequent time points (Fig. 5).

Monitoring the concentrations of RNA and proteins, we identified two significant differences between the concentration changes of these biomolecules in the nucleoli of HeLa and WI-38 cells. First, proteins and RNA dynamics in WI-38 cells have larger range of variations (Fig. 5). For example, in six randomly chosen HeLa cells RNA and proteins nucleolar concentrations during one hour changed within 19–33 mg/ml and 75–96 mg/ml, respectively.

While these ranges for six WI-38 cells are respectively 6–28 mg/ml and 44–86 mg/ml. The second difference discovered is that the concentration oscillation rates of the RNA and proteins concentration for HeLa cells are higher than for WI-38 cells. The average oscillation rate (frequency of variations occurring during the same time interval) for both concentrations in HeLa cells is ~4.3 per hour, while in WI-38 cells this parameter is ~3.0 per hour. Therefore, the analysis of RNA and protein concentration dynamics in the nucleoli reveals a higher oscillation frequency and a lower variations of the concentrations in HeLa as compared to WI-38.

## Discussion

Our study of protein, lipid and RNA concentrations in the organelles of live cultured cells, as well as monitoring of these concentration profiles in time, has unraveled substantial heterogeneity between the individual cells. These findings raise two intriguing problems: 1) Do individual organelles of the same type significantly differ in their functional activity, as is suggested by profound differences in the biomolecular concentration profiles? 2) Do the differences in molecular content of individual organelles indicate the heterogeneity of cellular functions? Furthermore, analysis of the obtained results is important for understanding the fuctions of specific organelles.

To answer these questions we analyzed the obtained concentration profiles in context of the organelle functions.

First, one-way ANOVA for biomolecular concentration data sets in two cell lines shows significantly different mean values for measurements of concentrations of macromolecules (Table 1S).

The concentration of proteins in the nucleolus, ER and mitochondria is significantly higher in HeLa cells than in WI-38 cells. The higher protein concentration in the nucleolus may be attributed to elevated rates of ribosome synthesis, and may reflect active recruitment of ubiquitous transcription and pre-rRNA processing machinery factors as well as multiple ribosomal proteins. Similarly, the higher concentration of proteins in the ER may reflect the overexpression of many genes, in HeLa cells.

The concentrations of RNA in the nucleolus and the mitochondria in HeLa cells are significantly higher than in WI-38 cells. As disscused above, higher amount of RNA in the nucleoli (Table 1S) point to more intense synthesis of rRNA and ribosome production in the HeLa cells. Similarly, the higher concentration of RNA in the mitochondria of HeLa cells indicate upregulation of mitochondrial genes transcription. However, the absolute concentration of RNA in the ER are similar for both the HeLa and WI-38 cells. This finding is surprising, since it does not correlate with the higher concentration of ER proteins found in HeLa cells (Table 1S). One possible interpretation of these data is that higher levels of protein sysnthesis in the ER of HeLa cells involve multiple translation cycles of the same mRNA sequence.

The BCA results clearly indicate the presence of lipids in the nucleoli of both WI-38 and HeLa cells. The accummulation of lipids in the nucleolus has been long established by the electron microscopy techniques^27–29^. Although the role of nucleolar lipids has yet to be defined, nucleoli in cancer cells, such as HeLa, harbor the enzymatic repair of peroxidized phospholipids^30^. Besides, the nucleolar phospholipids have been hypothesized to aid in the assembly of ribosomes^31^.

Our data demonstrate that the average concentration of lipids in nucleoli of HeLa cells is higher than in WI-38 cells. We propose that higher concentration of nucleolar RNA indicate upregulation of the ribosome synthesis, which further stimulates recruitment of phospoholipids to nucleolus, as discussed above.

Second, the correlation coefficients between biomolecular concentrations in different organelles of the same cell are different for WI-38 and HeLa cells. WI-38 cells show strong pairwise correlations between protein and RNA content of nucleolus and ER. In HeLa cells correlations between molecular profiles of these organelles were not pronounced. At the same time, HeLa cells demonstrate significantly higher correlation in concentrations of proteins and RNA in the mitochondria of the same cell (Fig. 4).

The data obtained during the monitoring of macromolecular profiles in time, suggest that variations between individual WI-38 and HeLa cells demonstrated different dynamics. The large fluctuations of molecular concentrations in WI-38 cells are suggestive of higher divergence in the functions of this nuclear organelle. Besides, high correlation coefficients between the proteins and RNA concentrations in nucleoli and ER (Fig. 4b), can further amplify the development of heterogeneity for this cell line over the time.

In conclusion, we demonstrate that measurements of absolute concentrations of macromolecules in cellular organelles using a microRaman-BCA approach opens up perspectives for a new “omics” direction. In parallel with subcellullar proteomics and other systemic studies of cellular structure, Ramanomics provides direct information on the macromolecular concentrations in discrete subcellular compartments of live cells. To the best of our knowledge, this study is the first report demonstrating the correlation between absolute concentrations of RNA and proteins in different organelles from the same cell. Analysis of microRaman-BCA generated data supports the concept that organelle-specific concentration profiles and correlation coefficient between the content of different organelles can serve as real-time quantitative markers to identify cellular heterogeneity.

## Materials and Methods

### Cell culture and fluorescent staining

HeLa and WI-38 cells were purchased from American Type Culture Collection. HeLa cells were continuously maintained and periodically renewed from the frozen stock. WI-38 cells used in study were in between 17th and 30 passages. Both cell lines were grown in glass bottom dishes (Mattek) and cultured in Advanced DMEM (Invitrogen), supplemented with 2.5% fetal calf serum (Sigma), 1% glutamax, 1% Antibiotic Antimycotic Solution (ThermoFisher Scientific) at 37 C in a humidified atmosphere with 5% CO_2_.

For routine control of the mycoplasma contamination, HeLa and WI-38 cells were fixed in ice cold methanol/acetic acid (3:1) mixture, stained by 10 μm Hoechst 33258 (ThermoFisher Scientific) and observed under the microscope. An absence of bacterial and fungal contamination was verified by periodic cultivation of the cells into Antibiotic-Antimycotic free media^32^.

To label mitochondria and ER, cells were incubated with MitoTrecker® Green FM (Invitrogen) and ER-Tracker^TM^Green (Invitrogen) for 30 minutes at a final concentration 500 nM and 1 µM, respectively. Then, cells were washed to remove unbound organelle probes and processed for imaging. For Raman measurements and fluorescent imaging, the cells were maintained in a Live-Cell^TM^ incubator (Pathology Devices) mounted on the microscope stage.

### Raman microspectrometry of cellular organelles

The confocal Raman microspectrometer is based on an inverted Nikon TE200 microscope equipped with single frequency laser diode (Ondax, 638 nm, 120 mW) excitation source, fiber-input MS3501i imaging monochromator/spectrograph (Solar TII), and HS101H – 2048/122-HR2 series CCD (Proscan) cooled down to −30 C. A 100^X^ Nikon oil-immersion objective lens with NA = 1.3 was used in the experiments. To enable signal acquisition in a confocal mode, a 100 micron pinhole was used. The confocal parameter was estimated to be ~1.8 micron by measurement of z-position dependence of Raman signal in thin (~200 nm) polystyrene film spin-coated on glass substrate. For spectral acquisition from specific organelles, the nucleoli were identified by the characteristic dense structure visible in the transmitted light, while the ER and mitochondria were labeled by green fluorescence organelles trackers, as described above. For visualization of the green fluorescence signal, 460–500 nm excitation and 510–560 nm emission band-pass filters were used. For acquisition of each spectrum the excitation laser was focused on an individual organelle. To ensure the absence of vibration, thermal drift, or other motion in our system during experiments, we visually verified the XYZ position of the cell before and after each measurement. While this experimental arrangement allows to pinpoint diverse subcellular structures, resolution of confocal Raman microscopy does not always ensure precise probing of pure organellar structures. This limitation is due to (a) potential difference between the confocal probing volume and the dimensions of organelles and (b) non-uniform structure of organelles. In particular, Raman scattering from biomolecules of fibrillar centers, dense fibrillar components and granular components in the nucleolus is averaged during acquisition, producing the nucleolar spectrum. Similarly, the Raman probing of the ER yields information on both, the ER lumen and surrounding cytosol. The contribution of cytosol is presented in Raman spectra of mitochondrion as well. Nevertheless, the differences of the Raman spectra in the probing volumes of distinct organelles are significant, and are, most likely, associated with biomolecular microenvironment of specific organelle.

All measurements were performed in live HeLa and WI-38 cell lines. To minimize potential phototoxic damage of cells during the experiments excitation wavelength, accumulation time and number of acquisitions were optimized for single cell measurements.

Accumulation time for spectra acquisition was 60 seconds. To increase accuracy, a series of three Raman spectra were sequentially acquired from the same organelle, and the spectra were averaged. In the time-lapse measurements of the same organelle, a single spectrum was acquired to avoid phototoxicity; the spectra measurements were spaced with 10 minute intervals. Under these conditions, experimental cells did not produce any visible changes in cellular morphology and showed no cytotoxicity by standard cell viability tests.

Preprocessing of Raman spectra including background subtraction, smoothing and baseline correction were included into BCA toolbox, developed in the framework of SBIR NIH R43GM116193 project. The preprocessing algorithm is briefly described, and examples of raw and preprocessed spectra, as well as residual spectra and subtracted background (Figs S1–S3), are shown in Supplementary materials.

### Biomolecular component analysis

For processing of multivariate spectral data obtained from the cell organelles, a Biomolecular Component Analysis (BCA) method, that provides detailed quantitative information on the biochemical constituents, was applied. This method was coded in the Matlab environment as “BCA toolbox”. In BCA approach, the concentration of RNA, DNA, proteins and lipids is obtained from the measured Raman spectra utilizing the linear combination modeling (LCM) as described in details in previous study^22, 26^. Briefly, Raman spectral concentration calibration was performed using bovine serum albumin, calf thymus DNA, *S. cerevisiae* RNA (Sigma), and bovine heart lipids (Avanti Polar Lipids). Unit weight of each biomolecular component was chosen to correspond to 100 mg/ml for proteins and 20 mg/ml for RNA, DNA and lipids concentrations. Raman profiles used for BCA components were measured using genomic DNA extracted from HeLa cells for DNA component and *S. cerevisiae* RNA for RNA component. The isolation was done with TRIzol kit (ThermoFisher Scientific) according to the manufacturer protocol. Lipid component was measured in lipid droplets of HeLa live cells. The HeLa protein profiles, obtained in previous Raman studies of HeLa cell line, were used as a protein component^22^. All component profiles were equalized to Raman intensities of unit weights, obtained during the concentration calibration procedure.

### Statistical analysis

Results are presented as mean of data from at least three independent measurements. Statistical analysis was performed by one-way ANOVA (p < 0.05) using Origin software. Details are provided in Table S1 (Supplementary Materials)

The number of measured cells: 143 HeLa and 68 WI-38 for nucleoli, 110 HeLa and 93 WI-38 for mitochondria, 39 HeLa and 39 WI-38 for ER. For time-lapse measurements 6 cells of both HeLa and WI-38 were chosen.

## Electronic supplementary material

Supplementary Information
