# Supplementary material for: Molecular profiling of single organelles for quantitative analysis of cellular heterogeneity
Source: Sci Rep. 2017 Jul 26;7:6512. doi: 10.1038/s41598-017-06936-z (PMC5529525; doi:10.1038/s41598-017-06936-z)
Supplement: Supplementary file 1 — Supplementary Information [file 41598_2017_6936_MOESM1_ESM.doc]

**Supplementary Materials**

Manuscript Title: Molecular profiling of single organelles for quantitative analysis of cellular heterogeneity

Authors: Andrey N. Kuzmin, Svitlana M. Levchenko, Artem Pliss, Junle Qu, Paras N. Prasad

Subtraction of background from Raman spectra. We developed an algorithm for automatic subtraction of background from the acquired spectra. To ensure the accuracy of this procedure, all background components (water, glass for glass-bottom Petri dish and fluorescence-baselane) were measured and smoothed to be used for background routine as input parameters (Figure S1). Routine testing demonstrated that at the distance of 1 μm or higher from the glass surface, background was confidently subtracted producing the high quality of the Raman spectra.

BCA tool preprocessing routine.

Preprocessing routine (BG.m) incorporates the following blocks:

- Loading of input data – measured spectrum, all of background and biomolecular components

- “Cosmic ray” removal;

- Savitzky-Golay smoothing (2nd order polynomial, 13 points);

- Spectrum adjustment for device spectral shift;

- Preliminary subtraction of all biomolecular and background weighted components from measured spectrum;

- Evaluation of residual spectrum;

- Second cycle of subtraction.

Supplemental Figure S1. Raman profiles of background components.

Raw spectra, preprocessed spectra, bacground profiles and residual spectra after background and model profiles subtraction.

Supplemental Figure S2. Example of Raman spectra of mitochondrion in HeLa cell: (a) series of raw spectra in the same organelle, (b) averaged raw spectrum, (c) modeled background, (d) preprocessed and residual spectra.

Supplemental Figure S3. Example of Raman spectra of nucleolus, mitochodrion and ER in WI-38 cell: (a) averaged raw spectra, (b) preprocessed and residual spectra.

Supplemental Table S1. One-way ANOVA (p<0.05) for sets of biomolecular concentrations in nucleolus, mitochodrion and RE for HeLa and WI-38 cell lines. Result Y/N means if mean values are significantly different.

|  |  | Proteins |  | RNA |  | Lipids |  |
| --- | --- | --- | --- | --- | --- | --- | --- |
|  |  | HeLa | WI-38 | HeLa | WI-38 | HeLa | WI-38 |
| Nucleolus | mean | 0.993 | 0.894 | 1.61 | 1.38 | 0.93 | 0.83 |
|  | variance | 0.018 | 0.02 | 0.12 | 0.12 | 0.05 | 0.05 |
|  | F | 23.34 |  | 20.83 |  | 8.78 |  |
|  | p | 2.62E-6 |  | 8.54E-6 |  | 0.003 |  |
|  | result | **Y** |  | **Y** |  | **Y** |  |
| Mitochondrion | mean | 0.64 | 0.70 | 0.58 | 0.48 | 2.07 | 1.98 |
|  | variance | 0.02 | 0.02 | 0.04 | 0.03 | 0.50 | 0.33 |
|  | F | 8.20 |  | 17.74 |  | 0.94 |  |
|  | p | 0.004 |  | 3.83E-5 |  | 0.33 |  |
|  | result | **Y** |  | **Y** |  | **N** |  |
| RE | mean | 0.64 | 0.71 | 0.61 | 0.56 | 0.82 | 1.00 |
|  | variance | 0.02 | 0.03 | 0.03 | 0.03 | 0.16 | 0.19 |
|  | F | 4.50 |  | 2.17 |  | 3.14 |  |
|  | p | 0.04 |  | 0.15 |  | 0.08 |  |
|  | result | **Y** |  | **N** |  | **N** |  |
